# Dataset on optimization of EDM machining parameters by using central composite design

**DOI:** 10.1016/j.dib.2019.01.019

**Published:** 2019-01-17

**Authors:** A. Soundhar, H. Abdul Zubar, Mohamed Thariq Bin Haji Hameed Sultan, Jayakrishna Kandasamy

**Affiliations:** a School of Mechanical Engineering, VIT University, Vellore, Tamilnadu, India; b Faculty of Engineering, King Abdulaziz University, Jeddah, Saudi Arabia; c Laboratory of Biocomposite Technology, Institute of Tropical Forestry and Forest Products (INTROP), Universiti Putra Malaysia, 43400 Serdang, Selangor, Malaysia

**Keywords:** Titanium alloys, Electrical discharge machining, Orthopedic implants, Response surface method

## Abstract

Newly prepared titanium alloy (Ti-13Zr-13Nb (TZN)) using powder metallurgy is considered in this investigation. Titanium alloys (TZN) are used in hip and knee replacement for orthopedic implants. Conventional machining, TZN alloys produce higher tool wear rate and poor surface quality, but this can be reduced by Electrical Discharge Machining (EDM) method. Moreover, EDM produce good biological and corrosion resistant surface. In this research, experiments were conducted by considering the influential process factors such as pulse on time, pulse off time, voltage, and current. The experiments were designed based on Response Surface Methodology (RSM) of face centered central composite design. Analysis of Variance (ANOVA) was conducted to identify the significance process factors and their relation to output responses such as Electrode Wear Rate (EWR), Surface Roughness (SR) and Material Removal Rate (MRR). Further, an empirical model was developed by RSM in order to predict the output responses.

## Specifications table

**Table:** 

Subject area	*Materials science*
More specific subject area	*Bio materials*
Type of data	*Text file, tables, graphs and figures*
How data was acquired	*Experimental investigation*
Data format	*Raw, calculated, analyzed, tabulated, contour plotted*
Experimental factors	*Input factors (pulse on time, current, pulse off time and voltage) and output responses are electrode wear rate, material removal rate and surface roughness.*
Experimental features	*In this prepared alloy specimens were machined by using electrical discharge machine with different operating conditions.*
Data source location	*School of Mechanical Engineering, VIT University, Vellore, Tamil Nadu, India*
Data accessibility	*Data is with the article.*
Related research article	Sengottuvel, P., S. Satishkumar, and D. Dinakaran. Optimization of multiple characteristics of EDM parameters based on desirability approach and fuzzy modeling. *Procedia Engineering,* 2013 [12].
	https://doi.org/10.1016/j.proeng.2013.09.185

## Value of data

• This data set has innovative information on EDM machining parameters for orthopedic implant applications.
• Data would be valuable to the researcher those who are doing research on optimization of EDM parameters for machining titanium alloy (TZN).
• To understand the empirical relationship between EDM input factors and output responses.

## 1 Data

The date presented information on alloy preparation, EDM machining parameter, material removal rate, electrode wear rate, surface roughness and optimum EDM machining parameter. Table 1 shows the characteristics of the powders used in the Ti-13Nb-13Zr alloy preparation. Chemical composition of the powders used in this experiment in Table 2. Table 3, Table 4, Table 5 show the EDM operating conditions, Levels of EDM Machining Parameters and Experimental designs data. ANOVA for Material Removal Rate, Electrode Wear Rate and Surface Roughness are presented in Table 6, Table 7, Table 8. Table 9 shows the optimum parameters for EDM machining parameters, while Table 10 represents predicted and observed values of TZN alloy.

**Table 1:** Characteristics of the powders used in the Ti-13Nb-13Zr alloy preparation.

**Characteristics**	**Ti**	**Zr**	**Nb**
Mean particle size (µm)	5	5	7
Morphology	Angular	Angular	Angular
Melting point (°C)	1670	1850	2468

**Table 2:** Chemical composition of the powders used in this experiment.

**Elemental powder**	**Impurity content (%)**				
	**N**	**O**	**C**	**Si**	**Fe**
Ti	0,872	0,349	0,073	0,025	0,040
Nb	0,038	0,620	0,020	–	0,040
Zr	0,080	0,450	0,028	–	0,030

**Table 3:** The EDM operating conditions.

Working conditions	Description
Electrode material	Graphite
Electrode polarity	Negativity
Specimen material	Ti-13Zr-13Nb
Working area (mm^2^)	50 × 50
Voltage	50–70 V
Current	8–16 A
Pulse ON time	6–10 µs
Pulse OFF time	7–11 µs
Dielectric fluid	Kerosene

**Table 4:** Levels of EDM machining parameters.

**Factor**	**Name**	**Units**	**Level**		
			**Low (− 1)**	**Center (0)**	**High (+ 1)**
A	Voltage	V	50	60	70
B	Current	A	8	12	16
C	Pulse ON time	µs	6	8	10
D	Pulse OFF time	µs	7	9	11

**Table 5:** Experimental designs and results.

**Run**	**Voltage (V)**	**Current (A)**	**Pulse on time (µs)**	**Pulse off time (µs)**	**MRR (g/min)**	**EWR (g/min)**	**SR (µm)**
1	70	8	10	7	0.23	0.003	11.558
2	60	12	8	9	0.473	0.007	14.717
3	60	12	8	9	0.441	0.005	14.867
4	70	8	6	7	0.0789	0.004	7.647
5	50	8	6	11	0.5075	0.0004	6.245
6	50	16	10	7	0.2574	0.0115	14.514
7	60	12	10	9	0.6162	0.007	13.608
8	70	16	6	11	0.086	0.004	10.168
9	60	12	8	9	0.4731	0.006	10.325
10	60	12	8	9	0.4482	0.008	15.851
11	70	8	6	11	0.4272	0.0004	9.04
12	70	16	10	11	0.616	0.0117	14.514
13	60	12	8	9	0.4623	0.017	16.24
14	60	16	8	9	0.5707	0.0101	11.728
15	60	12	8	9	0.4572	0.007	12.485
16	50	16	6	7	0.2193	0.008	10.008
17	60	12	8	7	0.322	0.008	12.512
18	50	8	6	7	0.099	0.0042	6.301
19	70	16	6	7	0.3206	0.0105	9.577
20	50	12	8	9	0.205	0.0076	12.629
21	50	8	10	11	1.051	0.0043	10.389
22	60	12	8	11	0.6305	0.0057	12.196
23	60	12	6	9	0.34	0.0044	7.545
24	50	16	6	11	0.0448	0.003	6.753
25	50	8	10	7	0.2004	0.0039	13.289
26	60	8	8	9	0.7129	0.0041	9.149
27	70	12	8	9	0.2412	0.0085	18.214
28	50	16	10	11	0.525	0.0107	16.758
29	70	16	10	7	0.4086	0.0133	14.814
30	70	8	10	11	1.0208	0.0036	14.322

**Table 6:** Analysis of variance for MRR.

**Source**	**Sum of squares**	**D*f***	**Mean square**	**F value**	***p*-value Prob > F**	**Status**
Model	1.741562	14	0.124397	720.0376	< 0.0001	Significant
A-voltage	0.005685	1	0.005685	32.90791	< 0.0001	
B-current	0.090923	1	0.090923	526.2797	< 0.0001	
C-Pulse ON time	0.436209	1	0.436209	2524.871	< 0.0001	
D-Pulse OFF time	0.427073	1	0.427073	2471.988	< 0.0001	
AB	0.014744	1	0.014744	85.34156	< 0.0001	
AC	0.002488	1	0.002488	14.39827	0.0018	
AD	0.003609	1	0.003609	20.88969	0.0004	
BC	0.00401	1	0.00401	23.21105	0.0002	
BD	0.339976	1	0.339976	1967.856	< 0.0001	
CD	0.195519	1	0.195519	1131.704	< 0.0001	
A^2	0.166072	1	0.166072	961.2574	< 0.0001	
B^2	0.070987	1	0.070987	410.8861	< 0.0001	
C^2	8.63E-06	1	8.63E-06	0.049924	0.8262	
D^2	1.68E-09	1	1.68E-09	9.7E-06	0.9976	
Residual	0.002591	15	0.000173			
Lack of Fit	0.001742	10	0.000174	1.025342	0.5225	Not significant
Pure Error	0.000849	5	0.00017			
Cor Total	1.744153	29

**Table 7:** Analysis of variance for EWR.

**Source**	**Sum of squares**	**d*f***	**Mean square**	**F value**	***p*-value Prob > F**	**Status**
Model	0.000317	14	2.2644E-05	3.4157886	0.0121	Significant
A-voltage	1.69E-06	1	1.6928E-06	0.2553569	0.6207	
B-current	0.000168	1	0.00016781	25.314124	0.0001	
C-Pulse ON time	4.99E-05	1	4.9933E-05	7.5323884	0.0151	
D-Pulse OFF time	2.84E-05	1	2.835E-05	4.2766323	0.0563	
AB	4.16E-06	1	4.1616E-06	0.6277725	0.4405	
AC	3.08E-07	1	3.0802E-07	0.0464652	0.8322	
AD	2.4E-07	1	2.401E-07	0.0362188	0.8516	
BC	1.57E-05	1	1.5682E-05	2.365551	0.1449	
BD	3.63E-06	1	3.629E-06	0.5474342	0.4708	
CD	1.93E-05	1	1.9272E-05	2.9071738	0.1088	
A^2	1.07E-06	1	1.0747E-06	0.1621113	0.6929	
B^2	2.43E-07	1	2.4255E-07	0.0365879	0.8509	
C^2	7.54E-06	1	7.5404E-06	1.1374552	0.3031	
D^2	7.03E-07	1	7.0318E-07	0.1060745	0.7492	
Residual	9.94E-05	15	6.6292E-06			
Lack of Fit	4.1E-06	10	4.104E-07	0.0215243	1	Not significant
Pure Error	9.53E-05	5	1.9067E-05			
Cor Total	0.000416	29

**Table 8:** Analysis of variance for SR.

**Source**	**Sum of squares**	**d*f***	**Mean square**	**F value**	***p*-value Prob > F**	**Status**
Model	255.1337	14	18.22384	5.138321	0.0016	Significant
A-voltage	9.342724	1	9.342724	2.634238	0.1254	
B-current	24.25329	1	24.25329	6.838362	0.0195	
C-Pulse ON time	141.5796	1	141.5796	39.91922	<0.0001	
D-Pulse OFF time	0.001513	1	0.001513	0.000426	0.9838	
AB	1.757613	1	1.757613	0.49557	0.4922	
AC	2.947231	1	2.947231	0.83099	0.3764	
AD	4.425764	1	4.425764	1.247871	0.2815	
BC	0.887835	1	0.887835	0.25033	0.6241	
BD	0.23064	1	0.23064	0.06503	0.8022	
CD	0.614264	1	0.614264	0.173195	0.6832	
A^2	17.36054	1	17.36054	4.894909	0.0429	
B^2	14.85477	1	14.85477	4.188393	0.0586	
C^2	13.19186	1	13.19186	3.719524	0.0729	
D^2	0.594352	1	0.594352	0.167581	0.6881	
Residual	53.19978	15	3.546652			
Lack of Fit	27.72855	10	2.772855	0.544311	0.8068	Not significant
Pure Error	25.47122	5	5.094245			
Cor Total	308.3335	29

**Table 9:** Optimum values of TZN alloy.

**Parameter**	**Optimum value**
Voltage [V]	59.23
Pulse current [A]	8
Pulse on time [μs]	6.55
Pulse off time [μs]	11

**Table 10:** Predicted and observed values of TZN alloy.

**Response**	**Goal**	**Predicted**	**Observed**	**Error (%)**
MRR [g/min]	Maximize	0.8024	0.7804	2.82
EWR [g/min]	Minimize	0.0011	0.0012	-8.3
SR [μm]	Minimize	6.2431	6.546	-4.851

## 2 Experimental design, materials and methods

### 2.1 Materials

The materials used in the investigations are titanium, niobium and zirconium powders. The characteristics of these powders are shown in Table 1.

### 2.2 Methods

The step by step processes selected for production of alloy materials (Ti-13Zr-13Nb) are Blended Elemental Method (BE), cold uni-axial pressing, cold isostatic pressing and vacuum sintering. Hydride-dehydrate process (HDH) is adapted to make the elemental titanium powders. In the hydride process, titanium powder is produced in the vertical furnace (at 500 °C and for 3 h) with positive pressure [1]. Subsequently at ideal temperature of a room, the hydride was granulated in a niobium container under vacuum of 10^−2^ Torr. In continuation, niobium and zirconium powders are produced in the similar process with the temperature range of 800 °C. The powder production is carried out using hydride process to produce increased sintering rate and reduced cost. The chemical compositions of the powders are tabulated in the Table 2 [2].

Initially, the powders are weighed in lot about 4 g and mixed at time interval of fifteen minutes using a double-cone blender. After mixing of powders, cold uniaxial pressing process is performed on the powders at 60 MPa in cylindrical (15 mm diameter) steel die without lubricants. Subsequently, cold isostatic press is used to pressurize the specimen at 350 MPa for duration of 30 s. The specimens are encapsulated under vacuum of 10^−2^ Torr in flexible rubber molds. Thermal Technology equipment was adapted to conduct the sintering process in niobium container with vacuum of 10^−7^ Torr and sintering temperatures were kept around 900–1500 °C with heating rate of 20 °C/min. Later arriving at the low temperature, specimens were kept at the selected temperature for an hour and subsequent to that, furnace is cooled to room temperature. Tradition methods were utilized to prepare the metallographic specimens [3].

The work materials (TNZ alloys) were used with the dimensions of 20 mm diameter and length of 35 mm and diameter of 10 mm graphite electrode (for higher MRR and lower EWR) was applied in the EDM. Thirty experiments (Table 5) was carried out using die-sinking EDM (Grace D-6030S). Maximum movement of *X*, *Y* and *Z* axis are 300, 250 and 300 mm respectively. The working conditions of EDM machine are shown in Table 3. The impulse jet flushing system using commercial grade kerosene (the dielectric fluid) was utilized to blush off the foreign contaminants from the igniting region in the EDM [4]. All the specimens are machined using EDM within 20 min. The electrode wear rate and material removal rate is computed by using the weight variation of the work piece and electrode material earlier and later the machining by digital weighing scale (0.001 g precision). The MRR & EWR can be calculated as;.

MRR=Wi−Wf/ρx t where, *W_i_* = Initial weight of the work piece material, *W_f_* = Final weight of the work piece material, *ρ* = Density of the work piece material, *t* = Machining period = 20 min.

EWR=Wb−Wa/t where, *W_b_* = Weight of Graphite electrode material before machining, *W_a_* = Weight of graphite electrode after machining, t = machining period [5].

### 2.3 Experimental design

The experiment is designed based on face centered central composite design by using response surface methodology [6].

#### 2.3.1 Parameters and levels

EDM operating parameters such as voltage, current, pulse on time and pulse off time have a prominent effect on the machining performance of titanium alloys [7]. Each parameter was placed at three equally spaced values generally coded as level −1 (minimum value), level 0 (central value), and level +1 (maximum value). The range of the input parameters pulse on time, current, pulse off time and voltage were selected as 6–10 µs, 8–16 A, 7–11 µs and 50–70 V respectively as shows Table 4.

#### 2.3.2 Design of experiments

RSM is an effective tool for developing, improving, and optimizing the processes by combining several input variables and assessing how their complex interactions affect the performance of the response variables [2], [5]. In this study the numbers of trials were designed by using Response Surface Methodology (RSM). The input portion of the Central Composite Design is a full factorial design with all the sequence of the parameters at three stage (high + 1 and low − 1) and collected of eight star points and six central points (coded level 0) [1]. The Central Composite Designs contains thirty experimental values at four input factors and after conducting experiments the output responses are shown in Table 5 [8].

## 3 Statistical analysis

Analysis of variance (ANOVA) was conducted with the aim of evaluating the influence of pulse on time, current, pulse off time and voltage. Table 6, Table 7, Table 8 shows the results of ANOVA for material removal rate, electrode wear rate and surface roughness. The analysis was carried out at 5% significance level and 95% confidence level [4].

### 3.1 Mathematical model for MRR, EWR and SR

The empirical models developed for output response material removal rate, electrode wear rate and surface roughness were evaluated by the F test. From the results the mathematical model is statistically valid to evaluate the output variable. The adequacy of model is tested using ANOVA analysis. It was found that the model F-ratio of 720.076 was obtained for material removal rate. However, the F-ratio of the lack of fit is 1.0253. It was found that the model F-ratio for electrode wear rate is 3.415 and the F-ratio of the lack of fit is 0.021. it was found that the model F-ratio for surface roughness is 5.1383 and the F-ratio of the lack of fit is 0.544. Hence the models are notable and their lack of fit is inconsiderable.

$\mathrm{MRR}=-8.10803+0.29398*A-0.13650*B-0.12425*C+0.10421*D+7.58906E\;-004*A*B+6.23438E-004*A*C-7.52188E-004*A*D\;-1.98516E-003*B*C-0.018218*B*D+0.027630*C*D\;-2.49610E-003*A^{2}+0.010543*B^{2}-3.68991E-003*C^{2}\;+8.60088E-004*D^{2}$

$\mathrm{EWR}=+0.016010-7.80062E-004*A-5.79770E-005*B+2.75685E-003*C\;-4.57785E-004*D+1.09375E-005*A*B-9.37500E-006*A*C\;-9.37500E-006*A*D+1.17187E-004*B*C-3.90625E-005*B\;*D+2.65625E-004*C*D+7.10526E-006*A^{2}-1.80921E-005\;*B^{2}-3.22368E-004*C^{2}-7.23684E-005*D^{2}$

SR=+22.89445−2.99977*A+4.27854*B+10.92146*C+0.37029*D−8.28594E−003*A*B−0.021459*A*C+0.026297*A*D+0.029445*B*C−0.015008*B*D+0.048984*C*D+0.025885*A2−0.14965*B2−0.56411*C2−0.11974*D2

where *A* – Voltage *B* – Current *C* – Pulse on time *D* – Pulse off time.

### 3.2 Effect of process parameters on material removal rate (MRR)

**Fig. 1:**
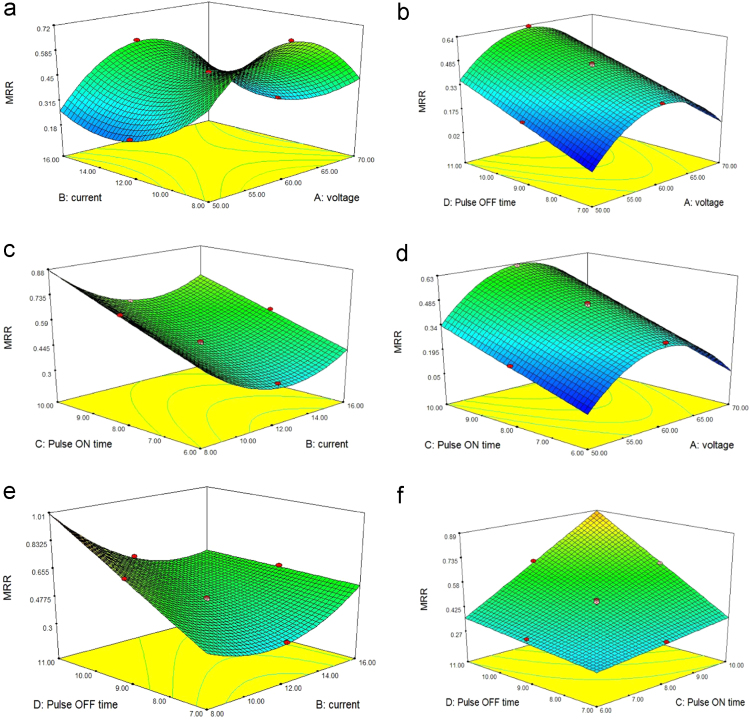
3D response surface plots showing (a) the impact of voltage and current (b) the impact of voltage and pulse off time (c) the impact of pulse on time and current (d) the impact of pulse on time and voltage (e) the impact of pulse off time and current (f) the impact of pulse off & pulse on time on material removal rate of TZN alloy.

When the pulse current and voltage` applied to the EDM machine was lesser, it reduces the output discharge energy. So lesser discharge energy will be applied into the machining zone, it causes poor material removal rate, fabricated chamber was narrower and the debris was easily evacuated from machined region. In reverse increase the voltage and current it produces higher the value of discharge energy.so wider chamber developed in the work specimen and also it distracts the electrical discharge, so it creates short circuit in EDM, results in small material removal rate. Hence optimum rate of current and voltage is essential to produce higher MRR. Fig. 1 displays the 3D surface plot for material removal rate in connection to the input factors such as current, pulse on time, voltage and pulse off time [1]. Fig. 1(a) displays the impact of current and voltage on material removal rate. When the voltage and current increases, It can be seen that the MRR increases significantly. When the pulse off time and voltage increases, considerably the MRR is also increased shown in Fig. 1(b). Fig. 1(c) and (d) shows the impact of pulse on time and current, pulse on time and voltage on MRR. It can be observed that the pulse on time increases, the MRR value is also increased. Fig. 1(e) display the impact of pulse off time and current on MRR. Fig. 1(f) depict the impact of pulse off and pulse on time on MRR. Thus the voltage, pulse on time, current and pulse off time are important parameters for material removal rate [7].

### 3.3 Effect of process parameters on electrode wear rate (EWR)

The electrode wear rate is a changing case which is altered by voltage, pulse on time, current and pulse off time with different input values. In EDM, the eroded materials from both work piece and tool, the damaged carbon particles from dielectric fluid may be accumulated on the tool face.it act as a protective layer in tool surface, that will help to reduce the tool wear rate.it can be achieved by higher value of pulse duration, lower value of pulse off time and pulse current.

**Fig. 2:**
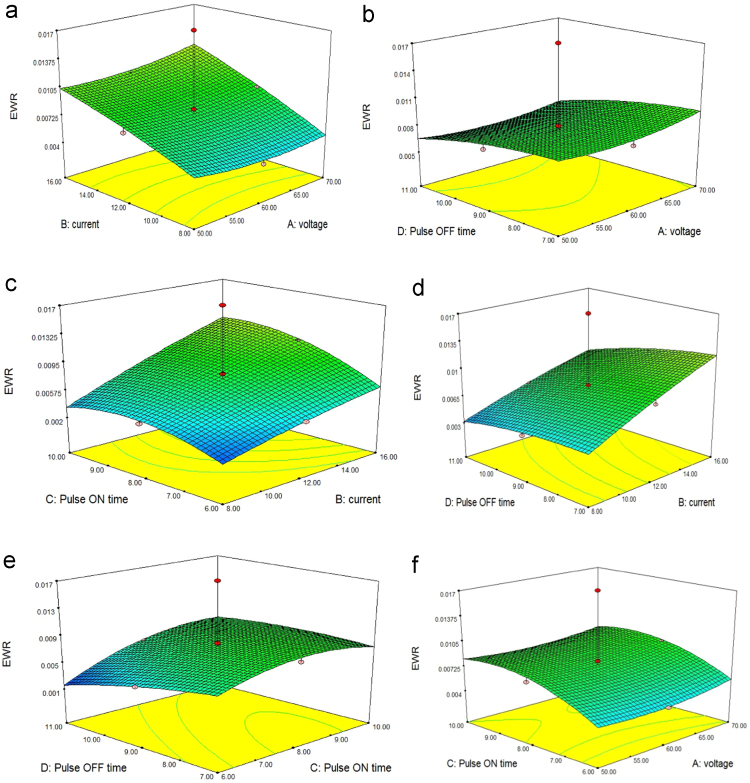
3D response surface plots showing (a) the impact of voltage and current (b) the impact of voltage and pulse off time (c) the impact of pulse on time and current (d) the impact of pulse off time and current (e) the impact of pulse on and pulse off time (f) the impact of voltage and pulse on time on electrode wear rate of TZN alloy.

Fig. 2 shows the relationship between input factors and electrode tool wear rate. Fig. 2(a) display the effect of voltage and current on EWR. When the pulse current increases, the value of EWR also increases. But either increases or decreases the value of voltage does not affect the value of EWR [9]. The same to be noted on Fig. 2(b) and (f). it means that voltage is inconsiderable factor for electrode wear rate. Whereas pulse on time, pulse off time and current is notable factors for electrode tool wear rate, it can be displayed in Fig. 2(c)–(e).

### 3.4 Effect of process parameters on surface roughness (SR)

**Fig. 3:**
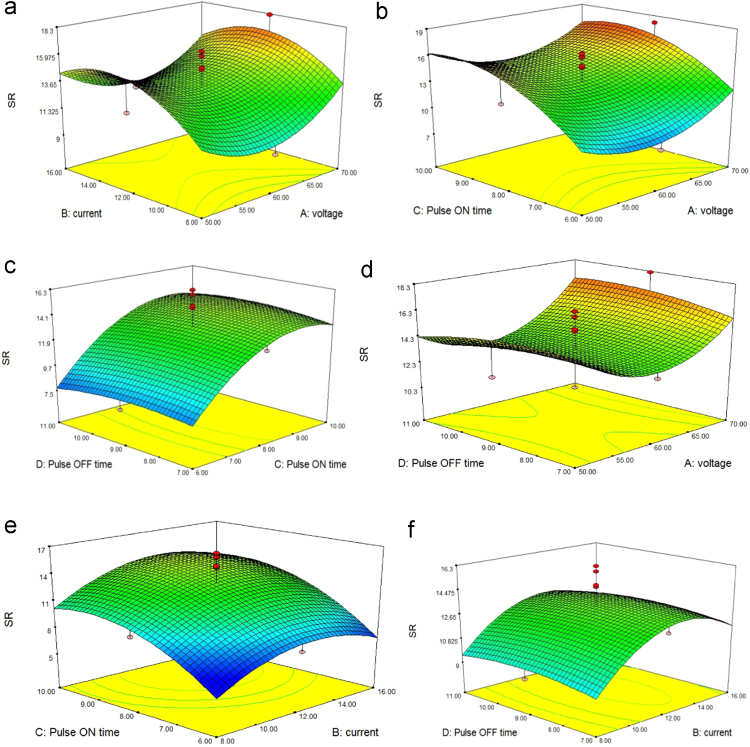
3D response surface plots showing (a) the impact of voltage and current (b) the impact of voltage and pulse on time (c) the impact of pulse on and off time (d) the impact of pulse off time and voltage (e) the impact of current and pulse on time (f) the impact of pulse off time and current on surface roughness of TZN alloy.

The high electrical discharge between tool and work piece produces crater wear on work piece surface which introduces poor surface finish.so optimum parameters to be needed to control the surface roughness. Fig. 3 shows the relationship between input variables (pulse on time, current pulse off time and voltage) and response (surface roughness).when the value of pulse on time and current rising simultaneously the surface roughness value also increases, it can be shown in Fig. 3(a), (b), (e) and (f) from Fig. 2(b) and (d), the roughness value is decreased up to 60 V and further rising the input voltage the value increases. Thus increasing or decreasing the value of pulse off time does not affect the surface roughness.so pulse off time is insignificant parameters for surface roughness [10].

## 4 Optimization and validation

The sequence has been valuated with the aid of Design Expert software version7.0. The desirability falls between zero and one. The solution with the highest desirability and close to one is chosen as the optimal setting and the validation experiments are conducted. The optimal parameters setting and the corresponding output values as shown in Tables 9 and 10.Through confirmatory experiments, it is observed that the absolute error between predictions and actual values fall within 10% [4], [11]. Thus the model can be effectively used to predict the EDM machining parameters.

The relative deviation was calculated with the following equation;

Relative deviation=Predicted value−Actual Value/Actual value*100
